# Alpha-1-Antitrypsin Deficiency in Children—Unmet Needs Concerning the Liver Manifestation

**DOI:** 10.3390/children11060694

**Published:** 2024-06-06

**Authors:** Joelle Lemke, Alexander Weigert, Soyhan Bagci, Mark Born, Rainer Ganschow, David Katzer

**Affiliations:** 1 Department of Pediatric Gastroenterology and Hepatology, University Hospital of Bonn Children’s Hospital, 53127 Bonn, Germanyalexander.weigert@ukbonn.de (A.W.); rainer.ganschow@ukbonn.de (R.G.); 2 Department of Neonatology and Pediatric Intensive Care Medicine, University Hospital of Bonn Children’s Hospital, 53127 Bonn, Germany; 3 Department of Pediatric Radiology, University Hospital of Bonn Children’s Hospital, 53127 Bonn, Germany

**Keywords:** alpha-1-antitrypsin deficiency, liver disease, children, predictors of poor outcome, icterus prolongatus

## Abstract

Objectives: This study aimed to analyse the clinical course of 45 children with severe alpha-1-antitrypsin deficiency (AATD) registered in our clinic to detect possible predictors of poor outcomes. Methods: The clinical and biological data of 45 patients with homozygous or compound heterozygous AATD were analysed. The data were collected retrospectively going back to 2005 and prospectively from May 2020 until October 2021. It was based on questionnaires, laboratory values, sonography, and biopsy findings. Liver disease was classified into four grades depending on the grade of liver disease: mild or no liver disease, moderate disease, severe disease, and liver transplantation. Results: Thirty-nine patients (86.7%) had a Pi*ZZ and five (11.1%) a Pi*SZ genotype. One patient showed a new, not-yet-described compound heterozygous genotype (Pi*Z + Asp95Asn). A total of 66.7% of the cohort showed mild or no liver disease, 20% moderate, and 13.3% severe. AATD was diagnosed in most cases because of liver abnormalities, such as the elevation of transaminases (42.2%). A total of 29.4% of the patients with neonatal icterus prolongatus developed severe liver disease, and 25.7% were born small for their gestational age (SGA). Diseases of the atopic type were reported in 47.4% of the cases. Conclusions: The presence of neonatal icterus prolongatus in the first weeks of life was significantly more likely in severe courses of liver disease (r = 0.371, *p* = 0.012). A tendency toward atopic comorbidity in AAT-deficient children needs to be further evaluated.

## 1. Introduction

Alpha-1-antitrypsin deficiency (AATD) is a genetic disorder predominantly characterised by liver and lung afflictions. While metabolic liver disease shows a peak prevalence in early childhood and after the age of 40, lung emphysema is not seen in children with AATD [1,2,3]. The disease is caused by a mutation in the *SERPINA1* gene inherited in an autosomal recessive pattern with a co-dominant expression of alleles. The *SERPINA1* gene codes the serine proteinase inhibitor alpha-1-antitrypsin (AAT), mainly synthesized by hepatocytes. Nearly 300 different mutations of the *SERPINA1* gene have been described [4]. S and Z alleles represent over 95% of the most frequent disease-associated mutations [5]. Europe counts a prevalence of approximately 1:3000 homozygous Glu342Lys-mutated individuals (Pi*ZZ) [6] and 1:1000 compound heterozygote Pi*SZ individuals [7].

A large variability in phenotypic expression in childhood favours a good disease course. This spectrum ranges from asymptomatic cases to transient neonatal cholestasis and may culminate in cirrhosis necessitating liver transplantation (LTx) [8]. The risk for a severe or deadly disease course is challenging to define, as most data originate from tertiary care centres with a selection bias [9,10]. The most valuable data arise from a Swedish epidemiological study from the 1970s identifying 127 patients with Pi*ZZ through the newborn screening of 200,000 children [11,12]. In this unbiased cohort, the risk of childhood cirrhosis-related mortality was reported to be 2–3%.

Unfortunately, predicting the individual course of the disease remains elusive, as no reliable predictor of adverse outcomes has emerged thus far. While some studies report on neonatal icterus prolongatus and neonatal cholestasis occurring more frequently in children with severe disease courses, many other factors, such as breastfeeding, gender, age at diagnosis, and more, have failed to correlate with disease progression consistently [13,14,15].

This study aimed to analyse the clinical course of a German cohort of 45 children with homozygous or compound heterozygous AATD who were medically supervised in our clinic to investigate possible predictors of poor outcomes.

## 2. Materials and Methods

Forty-five children of all ages with homozygous or compound heterozygous AATD (Pi*ZZ and Pi*SZ-genotypes) were regularly followed up at the Department for Paediatric Gastroenterology and Hepatology at Bonn University Hospital. All paediatric patients for whom homozygous or compound heterozygous AATD (Pi*ZZ, Pi*SZ or Pi*Z + other mutation) was detected before October 2021 were included. The patients were born between 1998 and 2019 and diagnosed with AATD between 2002 and 2020. The data were collected retrospectively from 2005 to May 2020 and prospectively from May 2020 to October 2021. The patients were seen at our clinic annually, bi-annually, or more often depending on the state and progression of their liver disease.

The following parameters were included: gender, genotype, age at evaluation, AAT serum level, age at diagnosis, mode of diagnosis, severity of liver disease, imaging data from sonography and biological data from liver biopsy, treatment and age at treatment start, breastfeeding, comorbidities (e.g., disease of atopic type), sibling history, laboratory results, and metric data. As liver biopsy plays a minor role in diagnosing AATD, it was not performed in all patients but only in those who showed progression of liver disease and those listed for liver transplantation.

The patients were divided into four groups with different liver disease stages, classified according to the status at the latest check-up. In the case of liver transplantation, the last data before liver transplantation were used. The different states of liver disease are defined in Table 1. The first group included all patients who underwent liver transplantation due to AATD. To be classified as severe liver disease, one of the following criteria had to be met: liver failure, persistent portal hypertension (PHT), or cirrhosis in biopsy or sonography. “Persistent” was defined as present on at least the last two check-up occasions and with an interval of a maximum of two years between the two measurements. PHT was evidenced by persistent platelets below 150,000/mm^3^, pathological coagulation (change in Quick/INR/Thrombin time, prothrombin time, or factor V), oesophageal varices, ultrasound/doppler ultrasound with portosystemic derivations, or reverse portal flow. Moderate liver disease was defined as persistent abnormal liver enzymes (ALT or GGT ≥ 2 N, more than twice the upper limit of normal) or persistent pathological signs in sonography (hepatomegaly/splenomegaly/liver parenchymal remodelling). The group of mild or no liver disease included all patients who did not meet any of the previous criteria and thus showed the following: absence of persistent PHT or cirrhosis of the liver, normal or almost normal liver biology in sonography, no persistent pathologies, and laboratory results with mildly abnormal or normal liver enzymes (ALT and GGT < 2 N, less than twice the upper limit of normal).

This study had to obtain no ethical approval, as only medically relevant data were collected for routine patient care. Questionnaires for detailed medical history were firmly implemented in our patient care and were not created especially for this study.

### Statistical Analysis

The statistical analyses were performed using SPSS 27.0 (SPSS Inc., Chicago, IL, USA). All data are presented as absolute number (percentage) or median (interquartile range (IQR)) if not further specified. Stepwise multiple linear regression analyses were used to analyse associations among the severity of liver disease, genotype, age at diagnosis, mode of diagnosis, and absence or presence of icterus prolongatus.

## 3. Results

The demographic and genetic characteristics of the studied population are summarised in Table 2. Most patients have the Pi*ZZ genotype (86.7%), and 11.1% have the Pi*SZ genotype. One patient showed a new, not-yet-described compound heterozygous genotype (Pi*Z + Asp95Asn) with an AAT serum level of 0.6 g/L. Our patients were mainly diagnosed due to liver abnormalities (an elevation of transaminases/coagulation disorder), icterus prolongatus, or familiar screening. A total of 44.7% of the patients developed icterus prolongatus or neonatal cholestasis.

Further insights into the characteristics of different liver disease groups are provided in Table 3. A severe disease course and the necessity for liver transplantation were exclusively observed in patients with the PiZZ genotype. Conversely, patients with PiSZ genotypes predominantly exhibited moderate or mild liver disease. A noteworthy proportion, comprising 64.3% of patients with at least moderate liver disease (groups 1 to 3) and 36.7% of those with mild or no liver involvement (group 4), received their diagnosis within the first year of life. Stepwise multiple linear regression analyses showed that exclusively icterus prolongatus was significantly associated with the development of severe liver disease (r = 0.371, *p* = 0.012). Remarkably, 83.3% of children who eventually required liver transplantation or experienced severe liver disease had a history of icterus prolongatus. Nevertheless, only 29.4% of children with neonatal icterus ultimately developed severe liver disease.

Table 4 provides a comprehensive overview of the laboratory findings. Liver transplant patients exhibited notably abnormal laboratory values concerning total bilirubin levels, particularly in the period shortly before liver transplantation. Regarding the evolution of laboratory values, of ALT and GGT in particular, many patients with pathological laboratory values demonstrated improvement or even normalisation over time. In the first six months of life, 75.0% (n = 16); at two years, 64.0% (n = 25); at five years, 47.3% (n = 19); and at eight years, 11.8% (n = 17) of the patients were concerned about ALT and GGT exceeding twice the upper limit of normal. Patients needing LTx did not normalise over time, and those with severe liver involvement all had elevated liver enzymes at least until five years of age. Children with moderate or mild/no liver disease were affected to a lesser extent by ALT and GGT exceeding twice the upper limit, with 60% (n = 5) and 71.4% (n = 7) in the first six months of life.

A total of 25.7% of the patients were born small for their gestational age. An analysis of z-values from regular body measurements (weight, height, BMI, and head circumference) indicated a weight deficit in children with severe liver disease in the first six months. However, this deficit tended to normalise as the children continued to grow.

## 4. Discussion

We confirmed previous reports of neonatal icterus prolongatus/cholestasis as one of the few suspected prognostic factors for poor outcomes [16,17]. No other prognostic factors could be detected in our cohort. To our knowledge, there are no previous data about the reported high frequency of allergic rhino-conjunctivitis or atopic dermatitis in children with severe AATD.

We observed a significant link between neonatal icterus prolongatus/cholestasis and the severity of liver disease in our study cohort. Among liver transplant recipients, 100% had experienced neonatal icterus prolongatus/cholestasis. Moreover, this association was also prominent among those with severe liver disease (75%) and moderate liver disease (71%), whereas it was less common among those with mild or no liver disease (28%). Our results align with similar published data, where the incidence of neonatal jaundice in severe liver disease ranged from 75% to 100% [11,16,18]. However, children with neonatal icterus prolongatus/cholestasis will not always progress to a severe disease course. Consistent with reports from other research centres, only 29.4% of individuals in our cohort developed severe liver disease or required liver transplantation [16]. This finding underscores the complexity of using neonatal icterus prolongatus/cholestasis as a singular prognostic factor for AATD-related liver disease. While it is an important marker, its predictive utility should be considered in the context of other clinical and genetic factors to provide a more comprehensive assessment of disease prognosis. This narrows the utility of icterus prolongatus and neonatal cholestasis as a good prognostic factor.

Interestingly, in only 11 children (24.4%), icterus prolongatus or neonatal cholestasis led to the diagnosis of AATD, although it was documented in at least 17 (44.7%) children. This finding supports our observation that AATD testing must be performed more on a low-threshold level. Every child with icterus prolongatus for more than three weeks, independent of the bilirubin differentiation, should be tested for AATD.

Regarding the laboratory values, an elevation of ALT and GGT exceeding twice the upper limit of normal within the first years of life does not indicate a poor prognosis for liver disease, as most values were normalised over time. Therefore, we agree that the clinical utility of simple AST, ALT, or GGT measures is limited to identifying patients with severe liver disease [19]. Nevertheless, children who ultimately presented with severe liver disease or necessitated liver transplantation exhibited this elevation in ALT and GGT levels from birth. This distinctive observation underscores the notion that while these laboratory values may not be predictive in all cases, they can serve as valuable indicators when combined with other diagnostic modalities, such as sonography.

Atopic disease emerged as a notable feature within our cohort, affecting 47.4% of the patients. This manifestation primarily manifested as allergies to pollen or grasses but also encompassed atopic dermatitis and allergic asthma. To contextualise our findings, we referenced national representative, population-based cross-sectional data from the KIGGS study (German Health Interview and Examination Survey for Children and Adolescents), which reported that 22.6% of German children and adolescents in the general population had been diagnosed with at least one physician-confirmed atopic disease [20]. Our data indicate that the prevalence of atopic disease within the AATD cohort exceeded that of the general population. It is worth noting that despite this heightened prevalence, we did not discern any correlation between the degree of liver involvement and the occurrence of atopic disease. Our findings align with prior research. Teckman et al. [10] previously reported a similarly elevated rate of asthma at 14%, while Sveger et al. [21] observed a 10% rate of asthma among AATD patients.

Moreover, Hoffmann et al. [22] highlighted a significantly higher incidence of atopic bronchial disease among heterozygote AATD Pi*MZ and Pi*MS individuals. They also discussed the potential pathogenesis underlying the heightened incidence of atopic disease in AATD. One hypothesis revolves around the role of serine proteases in allergen-induced histamine release. Mast cells, containing the serine protease inhibitor AAT, play a pivotal role in this process. It is conceivable that AATD could lead to increased histamine release by disrupting the regulation of serine proteases, thereby contributing to the higher prevalence of atopic disease in individuals with AATD.

Eden et al. reported a higher prevalence (48%) of atopic disease and upper airway obstruction among patients with AATD than patients (27%) with chronic obstructive pulmonary disease (COPD) [23]. This prevalence rate closely approximates the frequency of atopic disease observed within our cohort. Nevertheless, it is important to note that available data on the prevalence of allergic rhino-conjunctivitis or atopic dermatitis in children with severe AATD remain lacking.

Ursodeoxycholic acid (UDCA) was prescribed to 69% of patients in our cohort, closely mirroring the findings of Ruiz et al. in their French cohort [16]. In our cohort, the prescription rate was concordant with the severity of liver disease, with 100% in severe cases and 60% in patients with mild or no liver disease. The French cohort exhibited a similar trend but fewer prescriptions in patients with mild or no liver disease (35%). These findings collectively advocate for a proactive discussion regarding the early initiation of UDCA therapy in every patient diagnosed with AATD. While there is a valid argument for commencing treatment when liver disease progresses, there is also a compelling rationale for considering an early start to enhance the likelihood of a favourable disease course [24]. Supporting this recommendation, Lykavieris et al. [25] reported significant improvements in clinical status and liver test results among patients undergoing UDCA therapy. Although it is important to acknowledge that UDCA may not confer beneficial effects in cases of the most severe liver involvement, the prospect of early intervention remains reasonable.

This study has several limitations, including the absence of a control group, which complicates the determination of causal relationships. Additionally, due to the rarity of the disease, the cohort is relatively small, potentially limiting the generalisability of the results.

## 5. Conclusions

Our cohort sheds light on the significance of neonatal icterus prolongatus and neonatal cholestasis as potential indicators of a severe course of liver disease in AATD. It is essential to recognise that they do not serve as reliable standalone prognostic factors. Indeed, less than one-third of patients with neonatal icterus prolongatus or cholestasis progressed to a severe disease course. The role of laboratory parameters in assessing the disease course is crucial; however, our findings emphasise their limitations in identifying patients with severe liver disease, as these parameters often normalise over time. The intriguing relationship between AATD and atopic diseases suggests a complex interplay that merits in-depth exploration to unravel underlying mechanisms and inform potential therapeutic strategies. Moreover, it is of great importance to determine the pathomechanical reason why neonatal cholestasis often leads to a worse disease course. In this context, the mechanism of action of UDCA in the context of alpha-1 antitrypsin deficiency should also be explored to enable its more targeted and effective use.

## Figures and Tables

**Table 1:** Definition group of liver disease.

Group of Liver Disease	Criteria
1: Liver transplanted *	Liver transplantation due to AATD
2: Severe liver disease *	- Persistent ^1^ portal hypertension (PHT) ^2^ - Liver failure - Cirrhosis in biopsy or sonography
3: Moderate liver disease *	- Persistent ^1^ abnormal liver enzymes (ALT or GGT ≥ 2 N ^3^) - Persistent ^1^ pathological signs in sonography (hepatomegaly/splenomegaly/liver parenchymal remodelling)
4: mild or no liver disease *	- No persistent1 PHT ^2^ or cirrhosis of the liver - Normal or almost normal liver biology in sonography - No persistent pathologies and laboratory results with mildly abnormal or normal liver enzymes (ALT and GGT < 2 N ^3^)

AATD, alpha-1-antitrypsin deficiency; PHT, portal hypertension; ALT, alanine aminotransferase; GGT, Gamma-glutamyl transferase. * Group 1–3: ≥1 criteria had to be fulfilled, group 4: includes all patients who do not meet criteria of group 1–3 and thus all criteria from group 4 had to be fulfilled. ^1^ persistent defined as at least on the last two check-up occasions before evaluation and with an interval of maximum 2 years between the two measurements. ² PHT evidenced by persistent platelets below 150 G/l, pathological coagulation (change in Quick/INR/Thrombin time, prothrombin time, or factor V), oesophageal varices, ultrasound/doppler ultrasound with portosystemic derivations, or reverse portal flow. ^3^ More/less than twice the upper limit of normal.

**Table 2:** Characteristics of the studied population.

	n = 45
a. Basic Information	
Gender, n = 45 (%)	
- Girl	21 (47)
- Boy	24 (53)
Genotypes, n = 45 (%)	
- Pi*ZZ	39 (87)
- Pi*SZ	5 (11)
- Other compound heterozygosity with Z	1 (2)
Median (IQR) age at evaluation, y, n = 45	9.1 (5.2, 13.9)
b. Diagnosis	
Median (IQR) AAT serum level, g/L, n = 45	0.3 (0.3, 0.4)
Median (IQR) age at diagnosis, m, n = 44	13.0 (2.0, 40.3)
Age category at diagnosis, n = 44 (%)	
- ≤1 y	20 (45)
- >1 y	24 (53)
- Missing data	1 (2)
Mode of diagnosis, n = 45 (%)	
- Familial screening	10 (22)
- Icterus prolongatus/neonatal cholestasis ^a^	11 (25)
- liver abnormalities (elevation of transaminases/coagulation disorder)	19 (42)
- Others (ex., Growth abnormalities) and unknown	5 (11)
The severity of liver disease ^b^, n = 45 (%)	
- Liver transplanted	2 (4)
- Severe liver disease	4 (9)
- Moderate liver disease	9 (20)
- Mild or no liver disease	30 (67)
Median (range) age at biopsy, y, n = 10	2.25 (0.1–16.7)
Biopsy results, n = 9	
- Liver cirrhosis	3 (33)
- Liver fibrosis	4 (44)
- Other liver pathology	2 (22)
- No liver pathology	0
- Missing data	1 (11)
c. Medication	
UDCA, n = 45 (%)	31 (69)
No UDCA, n = 45 (%)	14 (31)
Median (IQR) age (m) at therapy start with UDCA, n = 31	18.5 (1.8–39.5)
d. Anamnesis, Comorbidities	
Icterus prolongatus/neonatal cholestasis ^a^, n = 38 (%)	17 (45)
Disease of atopic type, n = 38 (%)	18 (47)
- Bronchial asthma, n = 34 (%)	3 (9)
- Atopic dermatitis, n = 36 (%)	8 (22)
- Allergy to pollen/grasses, n = 37 (%)	16 (43)
Families within cohort	37
Sibling history, n = 38 (%)	
- Pi*ZZ or SZ sibling(s)	15 (40)
● Pi*ZZ sibling triplet	1
● Pi*ZZ sibling doublet	4
● Pi*ZZ + Pi*SZ sibling doublet	2
- No Pi*ZZ sibling(s)	16 (42)
- No sibling	7 (18)
- Missing data	7
SGA (small for gestational age) ^c^, n = 35 (%)	9 (26)

AAT, alpha-1-antitrypsin; UDCA, ursodeoxycholic acid. ^a^ Icterus prolongatus = neonatal icterus persisting for more than two weeks after birth, neonatal cholestasis = serum conjugated/direct bilirubin level > 1 mg/dL and >20% of the total bilirubin. ^b^ Liver transplanted and severe, moderate, mild, and no liver disease = as defined previously in Table 1. ^c^ Small for gestational age = weight below the 10th percentile for the gestational age.

**Table 3:** Characteristics within groups of liver disease.

	LTx ^b^ (n = 2)	Severe ^b^ (n = 4)	Moderate ^b^ (n = 9)	Mild or None ^b^ (n = 30)
a. Basic Information				
Gender				
- Girl/Boy, n	1/1	2/2	5/4	13/17
Genotypes, n (%)				
- Pi*ZZ	2 (100)	4 (100)	8 (89)	25 (84)
- Pi*SZ	0	0	1 (11)	4 (13)
- Other compound heterozygosity with Z	0	0	0	1 (3)
Median age at evaluation, y	4.6	13.1	2.9	9.5
b. Diagnosis				
Median AAT serum level, g/L	0.4	0.4	0.3	0.3
Median age at diagnosis, m	1.0	15.9	1.5	18.0
- Missing data, n	0	0	1	0
Age category at diagnosis, n (%)				
- ≤1 y/>1 y, n	2/0	2/0	5/3	11/19
- Missing data	0	0	1	0
Mode of diagnosis, n (%)				
- Familial screening	0	0	2 (22)	8 (27)
- Icterus prolongatus/neonatal cholestasis ^a^	2 (100)	1 (25)	3 (34)	5 (17)
- Liver abnormalities (elevation of transaminases/coagulation disorder)	0	3 (75)	2 (22)	14 (46)
- Others (ex., Growth abnormalities) and unknown	0	0	2 (22)	3 (10)
c. Medication				
UDCA/no UDCA, n	2/0	4/0	7/2	18/12
Median age at therapy start with UDCA, m	1.4	20.8	1.8	26.4
- Missing data	0	0	2	12
d. Anamnesis, Comorbidities				
Breastfeeding				
Yes/No, n	1/1	3/1	5/1	20/3
Missing data	0	0	3	7
Icterus prolongatus/neonatal cholestasis ^b^				
Yes/No, n	2/0	3/1	5/2	7/18
Missing data	0	0	2	5
Disease of atopic type, (%)				
Yes/No, n	1/1	1/3	3/3	13/13
Missing data	0	0	3	4
Subgroups of atopic type				
Bronchial asthma				
Yes/No, n	0/2	0/4	0/5	3/20
Missing data	0	0	4	7
Atopic dermatitis				
Yes/No, n	0/2	1/3	2/3	5/20
Missing data	0	0	4	5
Allergy to pollen/grasses				
Yes/No, n	1/1	0/4	3/3	12/13
Missing data	0	0	3	5
SGA (small for gestational age) ^c^, n (%)				
Yes/No, n	1/0	1/3	1/6	6/17
Missing data	1	0	2	7

LTx, Liver transplanted; AAT, alpha-1-antitrypsin; UDCA, ursodeoxycholic acid. ^a^ Icterus prolongatus = neonatal icterus persisting for more than two weeks after birth, neonatal cholestasis = serum conjugated/direct bilirubin level > 1 mg/dL and > 20% of the total bilirubin. ^b^ Liver transplanted and severe, moderate, mild, and no liver disease = as defined previously in Table 1. ^c^ Small for gestational age = weight below the 10th percentile for the gestational age.

**Table 4:** Maxima/minima of laboratory values within groups of liver disease.

Diagnostics	LTx ^a^ (n = 2)	Severe ^a^ (n = 4)	Moderate ^a^ (n = 9)	Mild or None ^a^ (n = 30)
Maximum ALT, U/L	328 (265–391)	152 (82–209)	115 (103–142)	95 (65–143)
Maximum GGT, U/L	1272 (1086–1458)	145 (84–539)	45 (38–503)	35 (23–71)
Maximum total bilirubin, mg/dL	6.7 (4.1–9.3)	0.7 (0.6–2.2)	2.8 (1.0–6.9)	0.5 (0.4–0.8)
Minimum platelets count, G/l	67 (53–81)	122 (89–182)	376 (275–387)	243 (205–293)

Data are presented as median (interquartile range (IQR)). LTx, Liver transplanted; ALT, Alanine aminotransferase; GGT, Gamma-glutamyl transferase. ^a^ Liver transplanted and moderate, mild, and no liver disease = as defined previously in Table 1.

## Data Availability

The original contributions presented in the study are included in the article, and further inquiries can be directed to the corresponding author/s.
